# Antiquity of Dentistry

**Published:** 1891-01

**Authors:** 


### Editorial, Etc.

ANTIQUITY OF DENTISTRY.—Dr. Talmage in a recent sermon remarks: "Dentistry, that we suppose one of the important arts discovered in recent centuries, is proven to be four thousand years old by the filled teeth of the mummies in the museums at Cairo, Egypt, and artificial teeth on gold plates found by Belzoni in the tombs of departed nations. We have been taught that Harvey discovered the circulation of the blood so late as the Seventeenth century. Oh, no! Solomon announces it in Ecclesiastes, where, first having shown that he understood the spinal cord, silver colored as it is, and that it relaxes in old age, "the silver cord be loosed," goes on to compare the heart to a pitcher at a well, for the three canals of the heart do receive the blood like a pitcher, 'or the pitcher be broken at the fountain.'

"What is that but the circulation of the blood found out twenty-six hundred years before Harvey was born? After many centuries of expl· ration and calculation astronomy finds out that the world is round. Why, Isaiah knew it was round thousands of years before, when in the Bible he said, 'The Lord sitteth upon the circle of the earth.' Scientists toiled on for centuries and found out refraction or that the rays of light when touching the earth were not straight but bent or curved. Why, Job knew that when ages before in the Bible he wrote of the light, 'It is turned as clay to the seal."

